# Supplementary material for: Disparate Roles of Oxidative Stress in Rostral Ventrolateral Medulla in Age-Dependent Susceptibility to Hypertension Induced by Systemic l-NAME Treatment in Rats
Source: Biomedicines. 2022 Sep 8;10(9):2232. doi: 10.3390/biomedicines10092232 (PMC9496567; doi:10.3390/biomedicines10092232)
Supplement: Supplementary file 1 [file biomedicines-10-02232-s001.zip › biomedicines-1849403-supplementary.pdf]

Disparate Roles of Oxidative Stress in Rostral Ventrolateral Medulla in Age-Dependent Susceptibility to Hypertension Induced by Systemic L-NAME Treatment in Rats

Yung Mei Chao[1], Hana Rauchová[2] and Julie Y.H. Chan[1,*]

Supplementary Figure 1

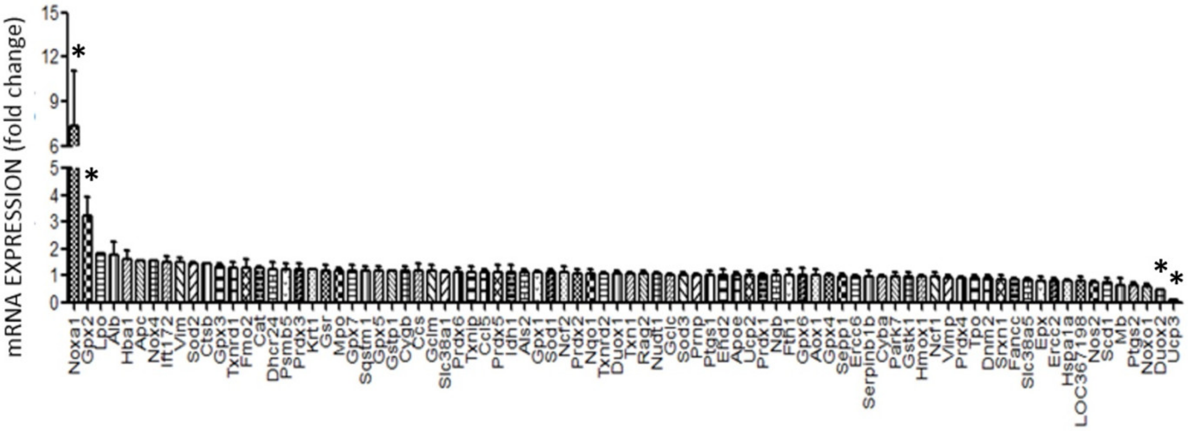

Figure S1. Differentially expressed oxidative stress-related genes in RVLM of adult rats. The oxidative stress-related transcripts were amplified by RT² Profiler PCR Arrays following the manufacturer's protocol. The microarrays include primer assays for 84 oxidative stress-focused genes, 5 housekeeping genes, a genomic DNA control, 3 wells contain reverse-transcription controls and 3 wells contain a positive PCR control (see table adopted from Cat. no. 330231 PARN-065ZA, Qiagen®). Tissues of bilateral RVLM from young (8 weeks) or adult (20 weeks) animals (n = 3 per group) were pooled for total RNA extraction. Samples were analyzed in triplicate, and relative quantification of gene expression was performed using the $2^{\Delta\Delta CT}$ method. Data are presented as mean mean ± SD, and astricks (*) demonte genes whose expression levels are at least two times different between adult and young rats.

Position	UniGene	GenBank	Symbol	Description
A01	Rn.202968	NM_134326	Alb	Albumin
A02	Rn.6408	NM_001013413	Als2	Amyotrophic lateral sclerosis 2 (juvenile) homolog (human)
A03	Rn.15681	NM_019363	Aox1	Aldehyde oxidase 1
A04	Rn.88057	NM_012499	Apc	Adenomatous polyposis coli
A05	Rn.32351	NM_138828	Apoe	Apolipoprotein E
A06	Rn.3001	NM_012520	Cat	Catalase
A07	Rn.8019	NM_031116	Ccl5	Chemokine (C-C motif) ligand 5
A08	Rn.12311	NM_053425	Ccs	Copper chaperone for superoxide dismutase
A09	Rn.100909	NM_022597	Ctsb	Cathepsin B
A10	Rn.5856	NM_024160	Cyba	Cytochrome b-245, alpha polypeptide
A11	Rn.105938	NM_130744	Cygb	Cytoglobin
A12	Rn.9470	NM_001080148	Dhcr24	24-dehydrocholesterol reductase
B01	Rn.11231	NM_013199	Dnm2	Dynamin 2
B02	Rn.162682	NM_153739	Duox1	Dual oxidase 1
B03	Rn.55542	NM_024141	Duox2	Dual oxidase 2
B04	Rn.16016	NM_001024897	Ehd2	EH-domain containing 2
B05	Rn.17695	NM_001107037	Epx	Eosinophil peroxidase
B06	Rn.74906	NM_001172809	Ercc2	Excision repair cross-complementing rodent repair deficiency, complementation group 2
B07	Rn.19370	NM_001107296	Ercc6	Excision repair cross-complementing rodent repair deficiency, complementation group 6
B08	Rn.10798	NM_012557	Fancc	Fanconi anemia, complementation group C
B09	Rn.3928	NM_144737	Fmo2	Flavin containing monooxygenase 2
B10	Rn.54447	NM_012848	Fth1	Ferritin, heavy polypeptide 1
B11	Rn.8365	NM_012815	Gclc	Glutamate-cysteine ligase, catalytic subunit
B12	Rn.2460	NM_017305	Gclm	Glutamate cysteine ligase, modifier subunit
C01	Rn.11323	NM_030826	Gpx1	Glutathione peroxidase 1
C02	Rn.3503	NM_183403	Gpx2	Glutathione peroxidase 2
C03	Rn.108074	NM_022525	Gpx3	Glutathione peroxidase 3
C04	Rn.3647	NM_017165	Gpx4	Glutathione peroxidase 4
C05	Rn.218434	NM_001105738	Gpx5	Glutathione peroxidase 5
C06	Rn.9852	NM_147165	Gpx6	Glutathione peroxidase 6
C07	Rn.4130	NM_001106673	Gpx7	Glutathione peroxidase 7
C08	Rn.19721	NM_053906	Gsr	Glutathione reductase
C09	Rn.109452	NM_181371	Gstk1	Glutathione S-transferase kappa 1
C10	Rn.87063	NM_012577	Gstp1	Glutathione S-transferase pi 1
C11	Rn.107334	NM_013096	Hba-a2	Hemoglobin alpha, adult chain 2
C12	Rn.3160	NM_012580	Hmox1	Heme oxygenase (decycling) 1
D01	Rn.1950	NM_031971	Hspa1a	Heat shock 70kD protein 1A
D02	Rn.3561	NM_031510	Idh1	Isocitrate dehydrogenase 1 (NADP+), soluble
D03	Rn.171849	NM_053792	Ift172	Intraflagellar transport 172 homolog (Chlamydomonas)
D04	Rn.31789	NM_001008802	Krt1	Keratin 1
D05	Rn.112309	XM_346005	LOC367198	Similar to Serine/threonine-protein kinase ATR (Ataxia telangiectasia and Rad3-related protein)
D06	Rn.60583	NM_001105829	Lpo	Lactoperoxidase
D07	Rn.40511	NM_021588	Mb	Myoglobin
D08	Rn.47782	NM_001107036	Mpo	Myeloperoxidase
D09	Rn.38575	NM_053734	Ncf1	Neutrophil cytosolic factor 1
D10	Rn.162331	NM_001100984	Ncf2	Neutrophil cytosolic factor 2
D11	Rn.64645	NM_033359	Ngb	Neuroglobin
D12	Rn.10400	NM_012611	Nos2	Nitric oxide synthase 2, inducible
E01	Rn.14744	NM_053524	Nox4	NADPH oxidase 4
E02	Rn.162651	NM_001100171	Noxa1	NADPH oxidase activator 1
E03	Rn.137764	NM_001106986	Noxo1	NADPH oxidase organizer 1
E04	Rn.11234	NM_017000	Nqo1	NAD(P)H dehydrogenase, quinone 1
E05	Rn.10669	NM_057120	Nudt1	Nudix (nucleoside diphosphate linked moiety X)-type motif 1
E06	Rn.30105	NM_057143	Park7	Parkinson disease (autosomal recessive, early onset) 7
E07	Rn.2845	NM_057114	Prdx1	Peroxiredoxin 1
E08	Rn.2511	NM_017169	Prdx2	Peroxiredoxin 2
E09	Rn.2011	NM_022540	Prdx3	Peroxiredoxin 3
E10	Rn.17958	NM_053512	Prdx4	Peroxiredoxin 4
E11	Rn.2944	NM_053610	Prdx5	Peroxiredoxin 5
E12	Rn.42	NM_053576	Prdx6	Peroxiredoxin 6
F01	Rn.3936	NM_012631	Prnp	Prion protein
F02	Rn.2	NM_001105727	Psmb5	Proteasome (prosome, macropain) subunit, beta type 5
F03	Rn.44404	NM_017043	Ptgs1	Prostaglandin-endoperoxide synthase 1
F04	Rn.44369	NM_017232	Ptgs2	Prostaglandin-endoperoxide synthase 2
F05	N/A	NM_001100528	Rag2	Recombination activating gene 2
F06	Rn.1023	NM_139192	Scd1	Stearoyl-Coenzyme A desaturase 1
F07	Rn.4197	NM_173120	Sels	Selenoprotein S
F08	Rn.1451	NM_019192	Sepp1	Selenoprotein P, plasma, 1
F09	Rn.137930	XM_225268	Serpinb1b	Serine (or cysteine) peptidase inhibitor, clade B, member 1b
F10	Rn.162022	NM_138832	Slc38a1	Solute carrier family 38, member 1
F11	Rn.81033	NM_138854	Slc38a5	Solute carrier family 38, member 5
F12	Rn.6059	NM_017050	Sod1	Superoxide dismutase 1, soluble
G01	Rn.10488	NM_017051	Sod2	Superoxide dismutase 2, mitochondrial
G02	Rn.10358	NM_012880	Sod3	Superoxide dismutase 3, extracellular
G03	Rn.107103	NM_181550	Sqstm1	Sequestosome 1
G04	Rn.2835	NM_001047858	Srxn1	Sulfiredoxin 1 homolog (S. cerevisiae)
G05	Rn.91199	NM_019353	Tpo	Thyroid peroxidase
G06	Rn.29777	NM_053800	Txn1	Thioredoxin 1
G07	Rn.2758	NM_001008767	Txnip	Thioredoxin interacting protein
G08	Rn.67581	NM_031614	Txnrd1	Thioredoxin reductase 1
G09	Rn.6300	NM_022584	Txnrd2	Thioredoxin reductase 2
G10	Rn.13333	NM_019354	Ucp2	Uncoupling protein 2 (mitochondrial, proton carrier)
G11	Rn.9902	NM_013167	Ucp3	Uncoupling protein 3 (mitochondrial, proton carrier)
G12	Rn.2710	NM_031140	Vim	Vimentin
H01	Rn.94978	NM_031144	Actb	Actin, beta
H02	Rn.1868	NM_012512	B2m	Beta-2 microglobulin
H03	Rn.47	NM_012583	Hprt1	Hypoxanthine phosphoribosyltransferase 1
H04	Rn.107896	NM_017025	Ldha	Lactate dehydrogenase A
H05	Rn.973	NM_001007604	Rplp1	Ribosomal protein, large, P1
H06	N/A	U26919	RGDC	Rat Genomic DNA Contamination
H07	N/A	SA_00104	RTC	Reverse Transcription Control
H08	N/A	SA_00104	RTC	Reverse Transcription Control
H09	N/A	SA_00104	RTC	Reverse Transcription Control
H10	N/A	SA_00103	PPC	Positive PCR Control
H11	N/A	SA_00103	PPC	Positive PCR Control
H12	N/A	SA_00103	PPC	Positive PCR Control
